# The Endermic Application of the Sulphate of Quinine

**Published:** 1846-08

**Authors:** H. V. Wooten

**Affiliations:** Lowndesboro’, Alabama


*The Endermic Application of the Sulphate of Quinine.* By H. V. Wooten, M. D., of Lowndesboro', Alabama.—In July, 1842, I was called to a lady affected with intermittent fever, of a rather grave type. She had suffered several paroxysms, and the fever now continued through the apyrexia. She was entering upon the fourth month of pregnancy, and suffering extreme irritation of stomach. A physician had been prescribing for her several days, during which time the sulphate of quinine had been given in combination with morphine and various other anodynes, and aromatics, but all had been instantly rejected. The bare effort to swallow produced retching, and it appeared that nothing could remain on the stomach, not even a small quantity of the ordinary secretions. Another paroxysm was expected in about four hours. I advised the administration of sulphate of quinine xx. grs., sulph. morph. ½ gr., in ℥i. gum arabic mucilage, to be thrown into the rectum, and repeated in three hours. I had found this mode of administration to answer an admirable purpose, in a few cases before, but the rectum seemed as irritable as the stomach, and the enema was promptly rejected. It was repeated and rejected three times, which I learned on my visit next day, and the paroxysm occurred at the regular hour. At every chill she was attacked with uterine pains so severe as to threaten abortion; and aside from that danger, her general condition presented rather an alarming aspect. The remedial powers of the quinine seemed to be absolutely necessary in the case, and the only remaining way of introducing it was by endermic application. It was about twenty-six hours to the time for the next paroxysm. There was already a blistered surface over the epigastrum about seven inches square. We cut a piece of adhesive plaster large enough to cover it, laid the plaster upon the bottom of a warm waiter, and when it was sufficiently soft, poured the quinine upon it, and rubbed it on with a spatula, until it formed a complete covering of the plaster, and applied it over the blister. We prepared another, twenty inches long, and three wide, which we applied to the spine, from the cervical vertebræ downwards, after sponging the skin with warm water. These plasters were kept carefully applied, (enough of the margin being left uncovered to adhere to the surface,) and nothing taken into the stomach but a few drops of water occasionally, when thirst rendered it absolutely necessary, until after the hour for the paroxysm, with the happiest effect. She had no chill, and as the chill was the cause of the uterine pains, and greatly aggravated the other symptoms, with it, she got clear of most of her distress, and improved very finely.

I have applied the sulphate of quinine endermically in four other cases, where, from various circumstances, I could not introduce it in a more direct way, and in every case with effect. I could detail them, but the practical result being so similar to the foregoing, it is unnecessary. I may mention, however, that in two cases the remedy was applied over a blister on the epigastrium, and in these it was successful in arresting the first paroxysm. In the two other cases, there was no blister, either on the epigastrium or spine; in one, it failed to arrest the first paroxysm, and succeeded on the second; in the other, it arrested the first, as in those cases where it was applied over a blister.—*Ibid.*

